# Comparison of thermal and athermal dynamics of the cell membrane slope fluctuations in the presence and absence of Latrunculin-B

**DOI:** 10.1088/1478-3975/accef1

**Published:** 2023-05-05

**Authors:** Srestha Roy, Rahul Vaippully, Muruga Lokesh, Gokul Nalupurackal, Vandana Yadav, Snigdhadev Chakraborty, Manoj Gopalakrishnan, Privita Edwina Rayappan George Edwin, Saumendra Kumar Bajpai, Basudev Roy

**Affiliations:** 1 Department of Physics, Indian Institute of Technology Madras, Chennai, India; 2 Department of Applied Mechanics, Indian Institute of Technology Madras, Chennai, India

**Keywords:** Membrane slope fluctuation, actin depolymerization, bending rigidity

## Abstract

Conventionally, only the normal cell membrane fluctuations have been studied and used to ascertain membrane properties like the bending rigidity. A new concept, the membrane local slope fluctuations was introduced recently (Vaippully *et al* 2020 *Soft Matter* **16** 7606), which can be modelled as a gradient of the normal fluctuations. It has been found that the power spectral density (PSD) of slope fluctuations behave as (frequency)^−1^ while the normal fluctuations yields (frequency)^−5/3^ even on the apical cell membrane in the high frequency region. In this manuscript, we explore a different situation where the cell is applied with the drug Latrunculin-B which inhibits actin polymerization and find the effect on membrane fluctuations. We find that even as the normal fluctuations show a power law (frequency)^−5/3^ as is the case for a free membrane, the slope fluctuations PSD remains (frequency)^−1^, with exactly the same coefficient as the case when the drug was not applied. Moreover, while sometimes, when the normal fluctuations at high frequency yield a power law of (frequency)^−4/3^, the pitch PSD still yields (frequency)^−1^. Thus, this presents a convenient opportunity to study membrane parameters like bending rigidity as a function of time after application of the drug, while the membrane softens. We also investigate the active athermal fluctuations of the membrane appearing in the PSD at low frequencies and find active timescales of slower than 1 s.

## 1 Introduction

Rheology of the cell membrane assumes great significance in cell locomotion, endocytosis, adhesion, differentiation and development [1–5]. Rheological parameters are also important in probing the health of the cell. For instance, cancer cells are softer and more elastic compared to healthy ones, which assists in intravasation [6] and spread throughout the body. However, the exact mechanism driving these elasticity changes is not known [7]. Cell membrane elasticity is also directly influenced in diseases like malaria [8] and sickle cell anaemia [9]. Membrane fluctuations are often responsible for transducing biophysical and biochemical signals between the cell and its environment and thus regulate cellular morphology and activities [10]. In view of all these facets, study of the cell membrane and the subsequent response to external stimuli attains enormous importance.

Membrane fluctuations are inherent in many active membrane processes, like ion-pump functioning, vesicle budding and trafficking [11–13] in living cells, not to mention the regular thermal fluctuations. Membrane dynamics is also influenced by the cyto-skeletal network [14–17]. The ERM class of protein cross linkers (ezrin, radixin, moesin) couples the cell membrane and cytoskeleton by binding the PIP2 domains of the membrane to the actin cortex [18–21]. Most of the active membrane processes are accompanied by reorganization of the actin cortex by depolymerization and subsequent polymerization. Treating the cell with drugs such as Latrunculin A/B, Cytochalasin A/B etc inhibits this polymerization process of actin cortex [22, 23]. Gradually the actin cortex is completely depolymerized which eliminates the link between membrane and the cytoskeleton rendering it more prone to flickering. Our knowledge of the mechanisms of the membrane processes shall be significantly improved upon learning the nature of active fluctuations [14, 21, 24] in these cell membranes.

Typically, normal membrane fluctuations have been studied to ascertain the rheological parameters in living cells [14]. These fluctuations are powered by thermal energy as well as by ATP consuming active processes. The temporal range of such fluctuations are quite broad, starting from very slow (10 s) actin waves that drive large wavelength fluctuations (100 nm to 10 *μm*) at cell edges and basal membrane [25–27], to relatively smaller amplitude ones (5 to 50 nm) which appear at the basal membrane [28, 29] and are mainly thermal in nature. We use a recently developed technique to place a particle on top of a cell membrane at locations away from the cell edges to find normal fluctuations after ensuring non-specific binding. Thus the unconfined apical surface of the cell can also be probed.

**Figure 1:**
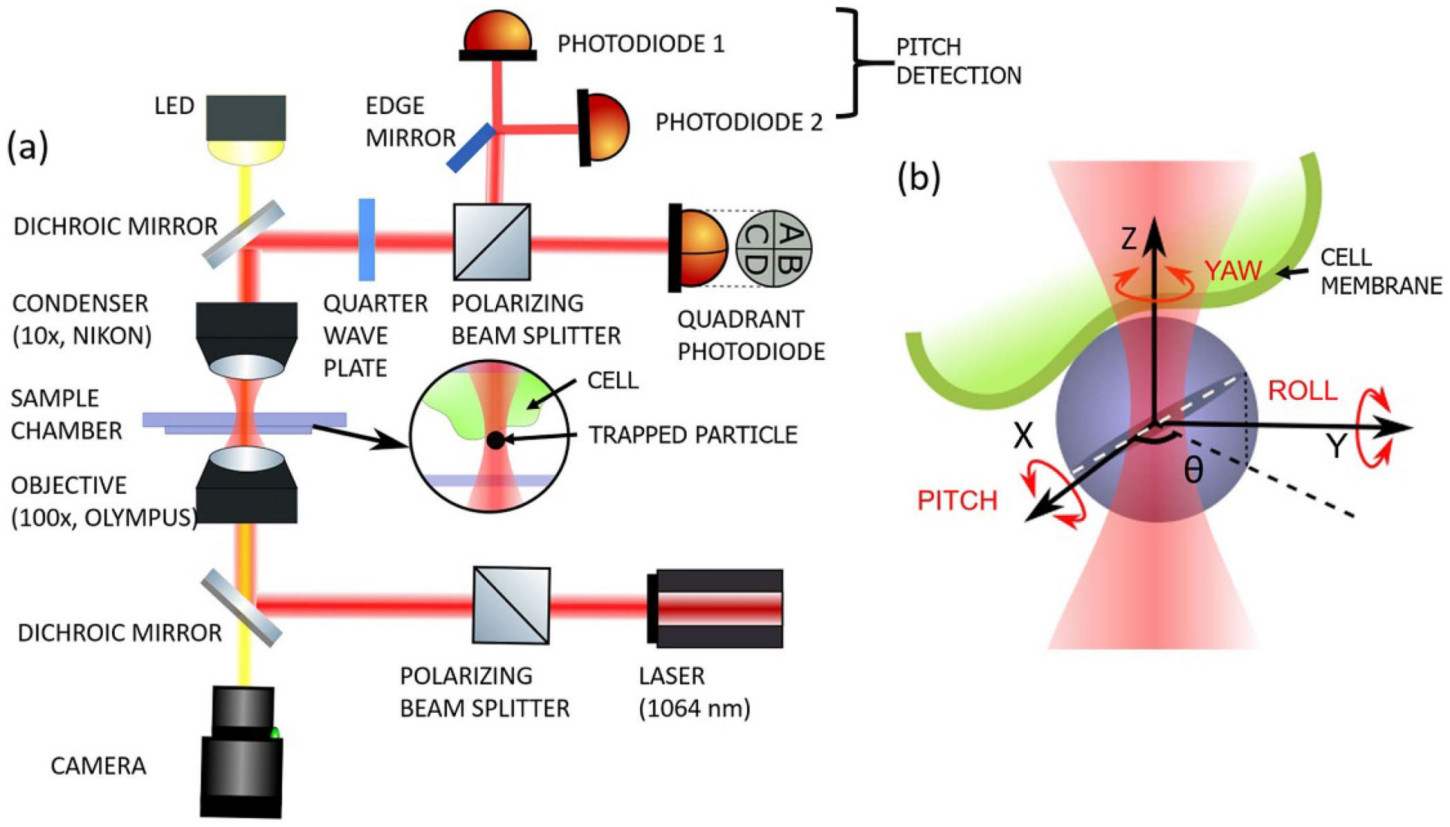
(a)Schematic diagram of Optical Tweezers set up. (b) RM-257 particle attached to cell membrane. The trapping beam having a Gaussian profile is shown in red. Rotational degrees of freedom of the spherical particle shown along *X*, *Y*, *Z* axes. The optic axis of the sphere is along the white dashed line. A change in slope of the point of contact causes a change in orientation of the optic axis.

In this technique, the particle is birefringent (has one refractive index along one direction different, with same refractive indices along the other two directions) [30] and has been placed with optical tweezers on the cell membrane. The pitch degree of rotational freedom [31] then provides information about the membrane slope fluctuations, as shown in figure 1. The particle turns in the out-of-plane sense due to the changes in local slope of the membrane.

We ascertained in [31] that the PSD for the normal membrane fluctuations exhibited a power law of (frequency)^−5/3^, as was expected from a free membrane. This result was surprising, as earlier measurements using flicker microscopy on the side of the cell yielded a power law of (frequency)^−4/3^, which then changed to (frequency)^−5/3^ due to the effect of depinning of the membrane from the cytoskeletal network upon application of the actin depolymerizing drug Latrunculin-B (Lat-B). We use this drug in this manuscript as well to ascertain the membrane slope fluctuations in our configuration. We find that the high frequency behaviour of the power spectral density (PSD) of the normal fluctuations still behaves as (frequency)^−5/3^ unless occasionally when (frequency)^−4/3^ is observed too. We also find that the PSD for slope angles as a function of frequency is exactly the same as that for the normal case without the drug both theoretically and experimentally. This can then be used to ascertain the softening of the membrane as a function of time under the influence of the drug. We further study the influence of the Lat-B on the active behaviour appearing at low frequencies of both the normal and the pitch PSD.

## 2 Theory

The PSD of an optically trapped particle in a viscous medium exhibits a Lorentzian behaviour as a function of frequency given by equation (1)

(1) $$PSD=\frac{A}{f^{2}+B}$$

where *B* is the square of corner frequency *f_c_* which is related to the optical trap stiffness as $\kappa =2\pi \gamma f_{c}$. Obtained PSDs are in Volts^2^ Hz^−2^ and are calibrated by a factor $\beta =\sqrt{\frac{k_{B}T}{\gamma A}}\cdot \gamma$ is the viscous drag coefficient near the cell membrane given by equation (2). where *γ*_0_ is the viscous drag coefficient far away from the surface, *ρ* is the particle radius and d is the distance between the centre of the particle and the surface.

(2) $$\gamma =\frac{\gamma_{0}}{1-\frac{5}{16}{\left(\frac{\rho }{d}\right)}^{3}+\frac{15}{256}{\left(\frac{\rho }{d}\right)}^{6}}$$

### 2.1 Passive thermal fluctuation

The cell membrane is modelled as an infinite plane membrane overlying a mesh of cytoskeletal filaments. The energy required to flicker a stretched bilayer membrane is given by the Helfrich Hamiltonian (equation (3)) [32].

(3) $$H[h(r)]=\int {\text{d}}^{2}r\{\frac{\sigma }{2}|(\nabla h(r){|}^{2}+\frac{\kappa }{2}{\left(\nabla^{2}h(r)\right)}^{2})\}$$

We have already shown the expected PSD for the slope fluctuations of a free membrane in [31]. However, the effect of pinning was not explored there. In the present manuscript, we extend the calculation to include pinning, i.e. the dynamics of the membrane in the presence of a hard surface. In order to account for the interaction, a harmonic potential term is included in the Helfrich Hamiltonian. The modified Helfrich Hamiltonian becomes equation (4).

(4) $$H[h(r)]=\int {\text{d}}^{2}r\{\frac{\sigma }{2}|(\nabla h{\left(r\right)}^{2}+\frac{\kappa }{2}{\left(\nabla^{2}h(r)\right)}^{2}+\frac{\lambda }{2}h^{2}(r)\}$$

where *σ* and *κ* are the surface tension and bending rigidity constant respectively, and *h*(*r*) denotes the membrane height. **r** is the two-dimensional position vector on the membrane. The last term included in the modified Hamiltonian (equation (4)) describes the effect of the pinning due to a hard surface placed in proximity to the lipid bilayer through a harmonic interaction potential. On depolymerizing the actin cortex with Lat-B, the coupling between cytoskeleton and membrane ceases to exist and the term *λ* becomes zero, when the membrane becomes depinned. The membrane fluctuations are characterised using the overdamped Langevin equation

(5) $$\frac{\partial h(r,t)}{\partial t}=-\int {\text{d}}^{2}r^{\prime }\frac{1}{8\pi \eta (r-r^{\prime })}\frac{\delta H}{\delta h(r^{\prime })}+\zeta (r,t)$$

where, (8*πηr*)^−1^ is the hydrodynamic interaction kernel and *ζ* (**r**, *t*) denotes the white noise term. We first analyse the general case, i.e. in presence of cytoskeletal confinement, as given in equation (4). Fourier transforming equation (5), we find

(6) $$\frac{\partial \hat{h}(q,t)}{\partial t}=-\frac{\xi (q)}{4\eta q}\tilde{h}(q,t)+\tilde{\zeta }(q,t)$$

where $\xi (q)=\frac{\kappa }{2}q^{4}+\frac{\sigma }{2}q^{2}+\gamma$. The solution of equation (6) is expressed as equation (7) where $\omega_{q}=\frac{\xi (q)}{4\eta q}$,

(7) $$\tilde{h}(q,t)=\int_{-\infty }^{t}{\text{e}}^{-\omega_{q}(t-t^{\prime })}\tilde{\zeta }(q,t^{\prime })\text{d}t^{\prime }$$

Since the transformed white noise term is delta-correlated in q- and time space,i.e.

(8) $$<\tilde{\zeta }(q,t)\tilde{\zeta }(q^{\prime },t^{\prime })>=(k_{B}T/4\eta q)\delta (q+q^{\prime })\delta (t-t^{\prime })$$

the height–height correlator turns out to be

(9) $$<\tilde{h}(q^{\prime },t^{\prime })\tilde{h}(q,0)>=\frac{k_{B}T}{4\eta q\omega_{q}}{\text{e}}^{-\omega_{q}t}\delta (q+q^{\prime })\delta (t-t^{\prime })$$

In order to introduce pitch fluctuations, we now define the slope-field (for small angle *θ*) on the membrane:

(10) $$\theta (r,t)≃\frac{h_{2}-h_{1}}{r_{2}-r_{1}}≃\frac{\partial h}{\partial r}$$

For fixed **r**, **q** **·** **r** = *qr*cos*ϕ* and *h*(**r**, *t*) is expressed as the inverse transform

(11) $$h(r,t)=\frac{1}{{\left(2\pi \right)}^{2}}\int {\text{d}}^{2}q\tilde{h}(q,t){\text{e}}^{-iqr\cos\phi }$$

The angle autocorrelator is then found to be

(12) $$A(t)\equiv \langle \theta (r,t)\theta (r,0)\rangle =\frac{\pi }{{\left(2\pi \right)}^{4}}\int \text{d}qq^{3}\frac{k_{B}T}{\kappa q^{4}+\sigma q^{2}+2\gamma }{\text{e}}^{-\omega_{q}t}$$

On Fourier transforming (*t* → *ω*), PSD $S_{\theta }(\omega )=\int_{-\infty }^{\infty }A(t){\text{e}}^{\text{i}\omega t}$ for *θ* is then found to be

(13) $$S_{\theta }(\omega )=\frac{k_{B}T}{{\left(2\pi \right)}^{3}4\eta }\int_{0}^{\infty }\text{d}q\frac{q^{2}}{\omega^{2}+\omega_{q}^{2}}$$

where $\omega_{q}=(\kappa /8\eta )q^{3}+(\sigma /8\eta )q+(\gamma /4\eta )q$. Define $\kappa_{1}=\kappa /8\eta ,\sigma_{1}=\sigma /8\eta \;\mathrm{and}\;\gamma_{1}=\gamma /4\eta$, so that

(14) $$S_{\theta }(\omega )=\frac{k_{B}T}{32\pi^{3}\eta }\int_{0}^{\infty }\frac{\text{d}qq^{4}}{\omega^{2}q^{2}+{\left(\kappa_{1}q^{4}+\sigma_{1}q^{2}+\gamma_{1}\right)}^{2}}$$

where $\beta_{1}=\frac{\sigma_{1}^{\prime }}{2}+\alpha ,\beta_{2}=\frac{\sigma_{1}^{\prime }}{2}-\alpha$ and $\alpha^{2}=\frac{\sigma_{1}^{\prime 2}}{4}-\gamma_{1}^{\prime }$. We assume that *α* is real and positive, which implies *β*_1_, *β*_2_ > 0 with *β*_2_ < *β*_1_. The integral in equation (14) may now be approximately expressed as the sum of three different integrals:

(15) $$S_{\theta }(\omega )≃\frac{k_{B}T}{(32\pi^{3}\eta }[I_{1}+I_{2}+I_{3}]$$

where

(16) $$I_{1}(\omega )≃\int_{0}^{\sqrt{\beta_{2}}}\frac{dqq^{4}}{\omega^{2}q^{2}+{\left(\kappa_{1}\beta_{1}\beta_{2}\right)}^{2}}I_{2}(\omega )≃\int_{\sqrt{\beta_{2}}}^{\sqrt{\beta_{1}}}\frac{\text{d}qq^{4}}{\omega^{2}q^{2}+\kappa_{1}^{2}q^{4}\beta_{1}^{2}}I_{3}(\omega )≃\int_{\sqrt{\beta_{t}}}^{\infty }\frac{\text{d}qq^{4}}{\omega^{2}q^{2}+\kappa_{1}^{2}q^{8}}$$

Note that we have made the following simplifications: in *I*_1_, we have assumed $q\ll \sqrt{\beta_{1}};\mathrm{in}I_{2}$ in *I*_2_, we have assumed $\sqrt{\beta_{2}}\ll q\ll \sqrt{\beta_{1}}$ and in *I*_3_, we assume $q\ll \sqrt{\beta_{1}}$ in the respective integrands. The three integrals in equation (16) can be easily evaluated, with the following results:

(17) $$I_{1}(\omega )=\frac{\beta_{2}^{3/2}}{3\omega^{2}}-\frac{\Delta^{2}\sqrt{\beta_{2}}}{\omega^{4}}+\frac{\Delta^{3}}{\omega^{5}}\tan^{-1}(\frac{\omega }{\Delta }\sqrt{\beta_{2}})$$

(18) $$I_{2}(\omega )=\frac{1}{\kappa_{1}^{2}\beta_{1}^{2}}\{\sqrt{\beta_{1}}-\sqrt{\beta_{2}}-\frac{\omega }{\kappa_{1}\beta_{1}}\times [\tan^{-1}(\frac{\kappa_{1}\beta_{1}^{3/2}}{\omega })-\tan^{-1}(\frac{\kappa_{1}\beta_{1}\sqrt{\beta_{2}}}{\omega })]\}$$

(19) $$I_{3}=\frac{1}{3\kappa_{1}\omega }[\frac{\pi }{2}-\tan^{-1}(\frac{\kappa_{1}\beta_{1}^{3/2}}{\omega })]$$

The dependence of *S_θ_*(*ω*) on the angular frequency *ω*, in the limit of small and large *ω* can be deduced as

(20) $$S_{\theta }(\omega )≃\frac{k_{B}T}{32\pi^{3}\eta }(A-B\omega^{2}+\ldots )(\omega \to 0)$$

(21) $$S_{\theta }(\omega )\sim \frac{k_{B}T}{3\pi^{2}\kappa \omega }(\omega \to \infty )$$

where the constants *A* and *B* are given by

(22) $$\begin{array}{c} A=\frac{\beta_{2}^{5/2}}{5{\text{Δ}}^{2}}+\frac{\sqrt{\beta_{1}}-\sqrt{\beta_{2}}}{\kappa_{1}^{2}\beta_{1}^{2}}+\frac{1}{3\kappa_{1}^{2}\beta_{1}^{3/2}}\\ B=\frac{\beta_{2}^{7/2}}{7{\text{Δ}}^{4}}+\frac{\sqrt{\beta_{1}}-\sqrt{\beta_{2}}}{\kappa_{1}^{4}\beta_{1}^{9/2}\sqrt{\beta_{2}}}+\frac{1}{9\kappa_{1}^{4}\beta_{1}^{9/2}} \end{array}$$

PSD in high frequency region (*ω* → ∞) can be expressed as a power law in terms of frequency *f* with $f=\frac{\omega }{2\pi }$ as

(23) $$PSD_{\theta }=Df^{\text{pow}\;}$$

Thus, there is no effect of the pinning due to a hard surface on the membrane, on expression for the pitch PSD. We use equation (23) to fit our experimental results. Estimates for bending rigidity are made by comparing equation (23) with equation (21). which gives $D=\frac{k_{B}T}{6\pi^{3}\kappa }$. As a special case, when the membrane fluctuations are unconstrained by the cytoskeleton due to actin cortex depolymerization, (i.e. *λ* = 0), we have *β*_2_ = 0. This leads to *I*_1_ = 0. Though the high frequency(*ω* → ∞) behaviour remains the same as in equation (21), the low frequency behaviour of the PSD changes as follows:

(24) $$S_{\theta }(\omega )=\frac{k_{B}T}{32\pi^{3}\eta }[\frac{4}{3\kappa_{1}^{2}\beta_{1}^{3/2}}-\frac{\pi }{2\kappa_{1}^{3}\beta_{1}^{3}}\omega +O(\omega^{2})](\omega \to 0,\gamma =0)$$

### 2.2 Active fluctuations

The cell membrane is also bound to various active proteins which induce conformational changes to the membrane [33, 34]. Local fluctuations induced by such active proteins are independent of each other and can be expressed a Langevin equation similar to equation (6).

(25) $$\frac{\partial \tilde{h}(q,t)}{\partial t}=-\frac{\xi (q)}{4\eta q}\tilde{h}(q,t)+{\tilde{\zeta }}_{\text{active}\;}(q,t)$$

Active forces due to action on active proteins is correlated in time as [35]

(26) $$<{\tilde{\zeta }}_{\text{active}\;}(0){\tilde{\zeta }}_{\text{active}\;}(t)>(q)={\left(\frac{F}{4\eta q}\right)}^{2}\frac{n}{2}{\text{e}}^{-\frac{\left|t\right|}{\tau }}$$

where n is the no. of active proteins acting in the region of interest, *η* is the coefficient of viscosity of the surrounding medium, F is the direct-force due to cytoskeleton on the membrane [12, 35] and *τ* represents the time scale over which the active proteins induce a fluctuation on the membrane. From equations (25) and (26), the height-height autocorrelation is found to be [35]

(27) $$<h|\omega ,q{|}^{2}>={\left(\frac{F}{4\eta q}\right)}^{2}\frac{\tau }{1+{\left(\tau \omega \right)}^{2}}\frac{n}{\omega^{2}+\omega_{q}^{2}}$$

If the local slope of the membrane changes by a small angle *θ*, it can be expressed as equation (10) [31].

(28) $$\theta (r,t)=\frac{\partial h}{\partial r}=\frac{1}{{\left(2\pi \right)}^{2}}\int {\text{d}}^{2}q(-\text{i}q\text{cos}\phi )\bar{h}(q,t){\text{e}}^{-\text{i}qr\text{cos}\phi }$$

(29) $$\theta (r,t)=\frac{-\text{i}}{{\left(2\pi \right)}^{2}}\int {\text{d}}^{2}q(q\text{cos}\phi )\tilde{h}(q,t){\text{e}}^{-\text{i}qr\text{cos}\phi }$$

(30) $$PSD_{\theta }=\iint <\theta (q,\omega )\theta (q^{\prime },0)>{\text{e}}^{-\text{i}q^{\prime }\cdot r^{\prime }}{\text{e}}^{-\text{i}q\cdot r}\times \delta (q+q^{\prime }){\text{d}}^{2}q^{2}q^{\prime }$$

invoking equation (27) in equation (30), the PSD for active slope fluctuations becomes,

(31) $${\mathrm{PSD}}_{\theta ,\;\text{active}\;}\approx {\left(\frac{F}{4\eta }\right)}^{2}\frac{n\tau }{\omega^{4/3}(1+{\left(\tau \omega \right)}^{2})}$$

## 3 Materials and methods

Our optical tweezers has been built around an OTK-B/M unit from Thorlabs, U.S.A. The main components of the optical tweezers set up are an oil immersion objective lens of working distance about 200 μm (100X, 1.3 NA, Olympus) and an air immersion condenser lens (10X, 0.25NA Nikon) which are aligned in a vertical column, as shown in figure 1. The objective lens tightly focus the 1064 nm trapping laser (Lasever from China) beam to a diffraction limited 1 μm spot. Whereas, the condenser lens collects the forward scattered light along with unscattered light and is also used to illuminate the sample chamber with white light from the LED as shown in figure 1(a). A polarising beam splitter passes linearly polarized light from a laser source towards the dichroic mirror which couples the laser into the vertical column onto the sample chamber and allows to image the same with CMOS camera. The sample to be studied is placed on the sample chamber which is positioned in between the objective and condenser lenses. The forward scattered light from the sample chamber is directed to the detection unit through a dichroic mirror. The detection unit consists of a quadrant photo diode (QPD) (Thorlabs) to detect the translations and it is aligned orthogonal to a pair of photodiodes (DET100A2, Thorlabs) which detect the rotational motion. The tracer particles that we use are liquid crystalline, birefringent RM 257 (Merck) particles, prepared using the standard protocol described in [36, 37]. When an RM 257 colloidal particle is confined in optical tweezers, its birefringent axis aligns along the polarization of the trapping beam, both in pitch and yaw sense [38–40]. If a well-linearly polarized light is used to trap particle, some amount of light will emerge from the dark-port of PBS, which accounts for orthogonal component of forward scattered light [41]. This is due to the internal structure of the liquid crystal directors of the particle, resulting in a four-lobe scatter intensity pattern in the orthogonal plane with respect to the incident polarisation. It has been demonstrated in [42] that when the particle rotates in the pitch sense, the distribution of light between the halves of the four-lobe becomes anisotropic. We use the same aspect to determine the pitch motion. We employ an edge mirror to divide the four lobes from the dark port into equal halves and send each halves to the photo detectors as shown in figure 1(a), where light from the other port is sent directly to the QPD. The voltage signals from the photodiodes are amplified further and interfaced by a data acquisition card (DAQ, National instruments) to the computer.

Rotational passive thermal fluctuations of these birefringent particles non specifically bound to the cell membrane of MCF-7 cells are exploited to measure the dynamics of the cell membrane fluctuation [31]. Human breast cancer cells, MCF-7 (obtained from National Center for Cell Science, Pune, India) were maintained in Dulbecco’s Modified Eagle’s Media (DMEM) supplemented with 10% fetal bovine serum (Gibco) and 1% penicillin-streptomycin (Gibco) to prevent contamination by any bacterial growth. For the experiment, the cells were grown on gelatin coated glass coverslips (50 mm × 20 mm). Initially, the glass coverslips were cleaned thoroughly in detergent followed by wash in concentrated HNO_3_. The coverslips were sterilized by UV treatment for 1 h. For gelatin coating, a droplet of 0.1% gelatin solution was added to the center of the coverslip and incubated at 37 °C for 1 h. The coverslip is then washed using 1X PBS and 20 *μ*l of cell suspension (10^5^ cells/ml) was added to the center of the coverslip. The cells were incubated at 37 °C and 5% CO_2_ for 1 h to allow the cells to settle and attach to the surface. CO_2_ along with H_2_CO_3_ present in the nutrient medium assists in maintaining the pH level within the range of 7.2–7.4(slightly alkaline) which is essential for keeping the cells alive. After the cells are attached, 500 *μ*l of fresh media is added. For probing the membrane rheology, 5 *μ*l of 1 ± 0.1 *μ*m diameter RM-257 particles (5 *μ*l of stock suspended in 100 *μ*l of sterile serum free media) is added to the cells about 40 min before starting the experiment to avoid endocytosis of the particles.

The experiment is carried at a constant temperature of 25 °C in the room maintained with an air conditioner. RM-257 beads are trapped on the cell membrane and the signal from the pair of photo diodes is amplified with current amplifiers and transmitted to a computer via Data Acquisition Systems (DAQ, National Instruments) having a sampling rate of 40 kHz. PSDs are recorded every 5 s and averaged over ten such PSDs.

## 4 Results and discussions

The cell membrane is often divided into subdomains each differing from the other in certain properties. Conformational changes in a cell such as vesicle budding, phagocytosis, cell fission, budding are often accompanied by changes in curvature and bending which are localised to that particular subdomain of the membrane [3]. We have measured local slope fluctuations of the membrane of 20 different cells treated with the drug Lat-B.

Lat-B is known to depolymerize the actin cortex. The thermal passive fluctuations of the membrane is picked up by the birefringent particle attached to the membrane.

**Figure 2:**
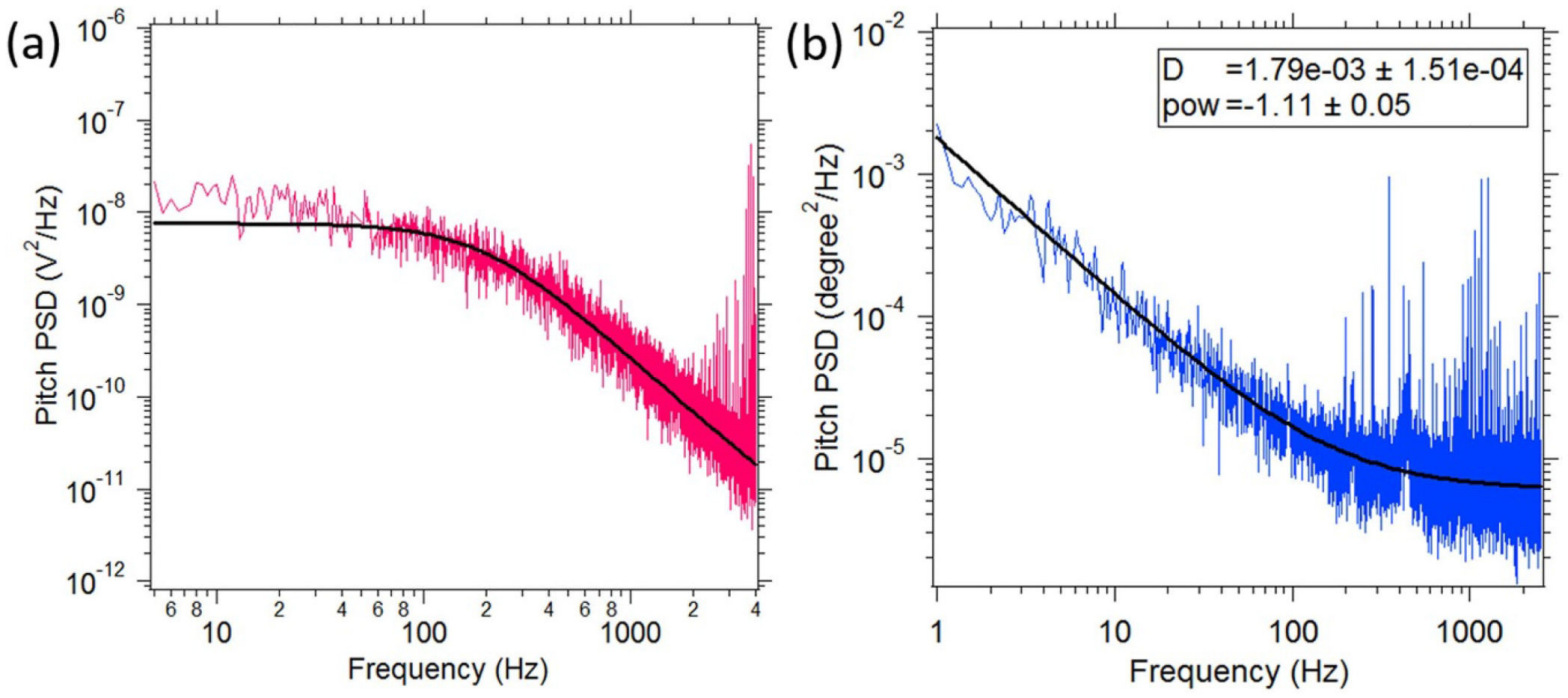
(a) Power spectral density recorded for the pitch motion of a birefringent particle trapped in water shows a Lorentzian nature as a function of frequency. The experimental data is shown in pink and the fit with equation (1) is shown with black solid line (b) Power spectral density for pitch motion of a particle non specifically bound to a cell membrane treated with Latrunculin-B has been shown as a function of frequency The experimental data (blue) has been fitted to equation (23) shown by black solid line.

**Figure 3:**
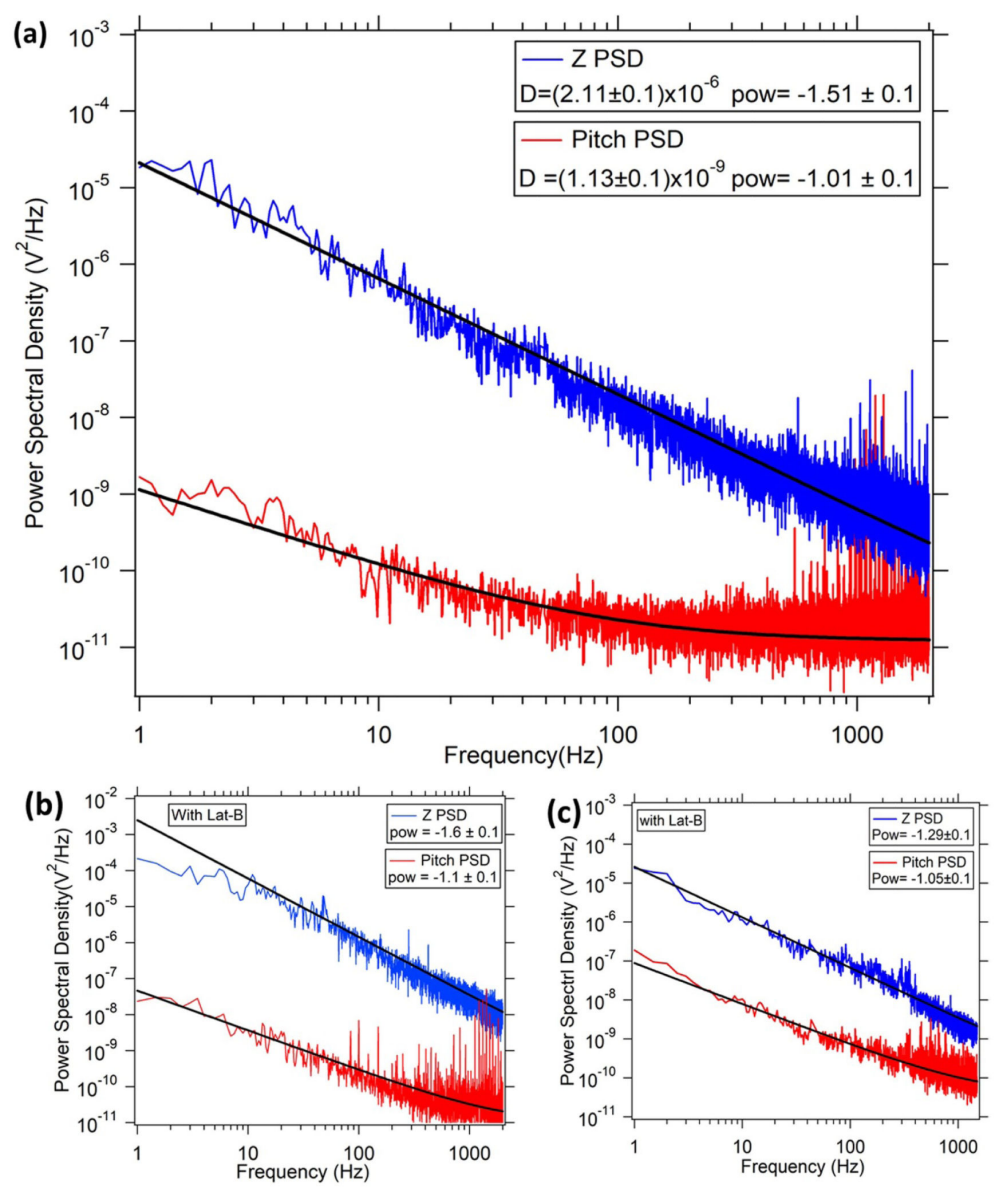
(a) PSD for membrane fluctuations of a cell without any drug (control experiment), for both Z (blue) and Pitch (red) PSD. (b) and (c) cell treated with Lat-B. Thermal fluctuations in Z in (b) show an exponent ∼1.6 but (c) shows an exponent ∼1.33 with *ω* (which sometimes happens particularly late after adding lat (B) whereas PSD for slope fluctuations vary as ∼*ω*^−1^ always.

The PSD along *Z* axis shows the normal fluctuation of the membrane whereas the PSD for out of plane pitch rotation shows the slope fluctuations. We show a pitch PSD in figure 2, while a set of typical normal PSD's in figure 3, where the presence of the Lat B influences the amplitude at high frequencies, but yet the power law remains (frequency)^−5/3^. This power law for cellular apical membrane is expected since it is not bounded by any surface. However, it is different from flicker microscopy results where the power law in the normal conditions is (frequency)^−4/3^, which then changes to (frequency)^−5/3^ in the presence of the actin-depolymerizing drugs [43, 44]. We show three cases in figure 2. First, when there in no Lat-B, where the normal PSD has an exponent of −1.5 ± 0.1. In the second case, we have Lat-B but the normal exponent yields −1.6 ± 0.1, while in the third case the normal exponent is −1.3 ± 0.1 which is observed sometimes, particularly late after placing the cell on the microscope. In all cases, the pitch exponent is consistent with −1.

**Figure 4:**
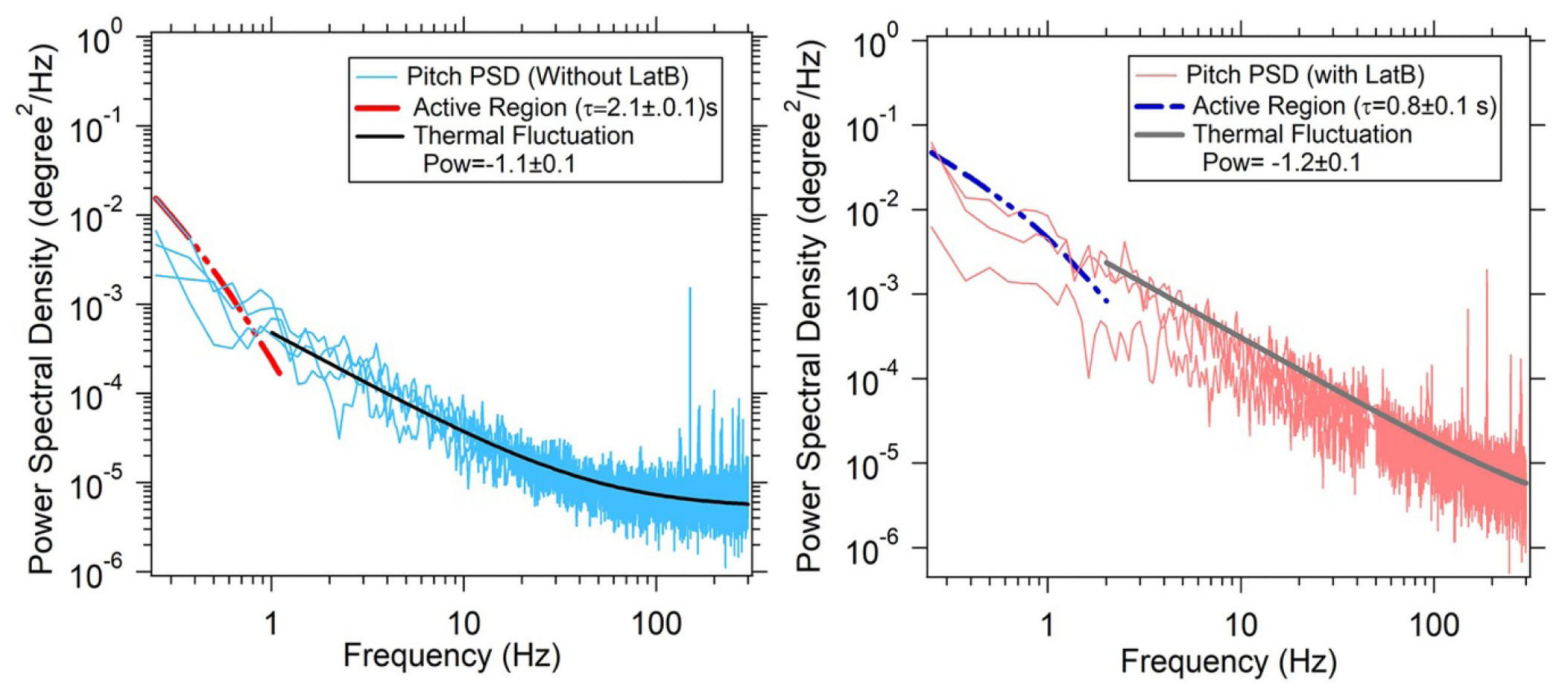
Pitch PSD (with Latrunculin B and without the drug in normal conditions) fitted to active fluctuations at low frequency and thermal fluctuation at high frequency.

We also fit the low frequency behaviour of the PSD to active laws, expected from equation (27), as shown in figure 4, on the lines of [45]. We do not find any convincing region between 0.3 Hz and 10 Hz which would imply activity neither in the normal case, nor in the case with lat-B present. Thus we find that the actin dependent activity does not appear above 1 Hz in PSD. Just to highlight the effect, we fit the equation (27) to the PSD's and find a time constant *τ* of about 1 s. However, the active fit itself is not convincing. This is consistent with expectations that active fluctuations due to actin polymerization possibly happen at the timescale of min (i.e. 0.1 Hz or lesser in PSD).

**Figure 5:**
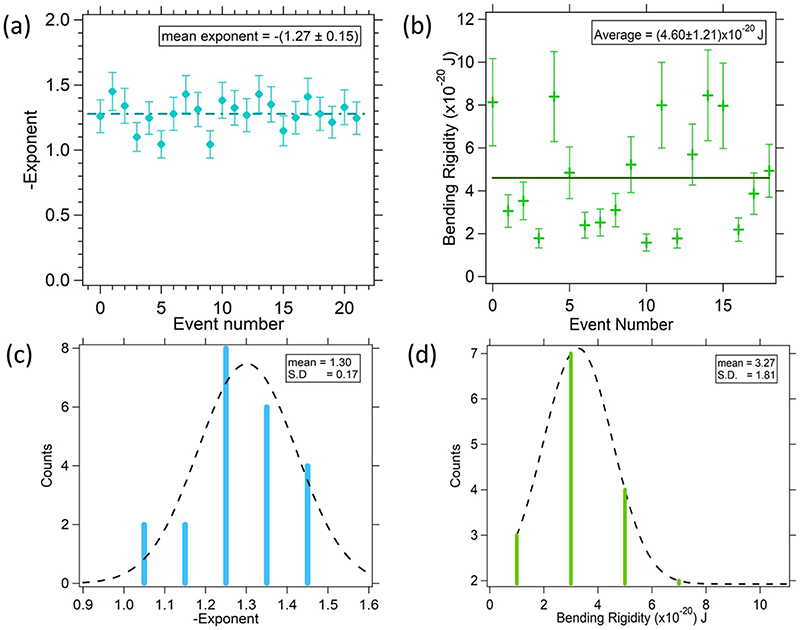
(a) Exponents obtained from PSDs for pitch motion fitted to equation (23) for different particles on the membrane of different cells. The exponent and coefficient obtained with every PSD fit has been called an event. Experimental data points are shown in red, black solid line corresponds to the mean value. The average exponent is −1.27 within an error of *±*0.15 which closely follows the theoretical value of − 1. (b) Bending rigidity *kappa* measured for Lat-B treated cells yields a mean value of 4 × 10^–20^ J. (c) Distribution of exponents fitted to a Gaussian (d)Distribution of measured *κ* values fitted to Gaussian.

A typical normal and pitch PSD without Lat-B is shown in figure 3(a). It shows a power law behaviour as a function of frequency having an exponent of −1.11. The exponents obtained from power law fitting of the PSDs as a function of frequency (equation (23)) for different events is shown in figure 5(a). The best fit with a straight line having 0 slope yields a mean value of −1.27 ± 0.15 which is in accordance with the with the theoretical value of −1 (equation (21)). However, the higher value of exponent could be a result of the finite size of the particle [46, 47]. The slope fluctuations show a consistent inverse law behaviour in both the cases.

**Figure 6:**
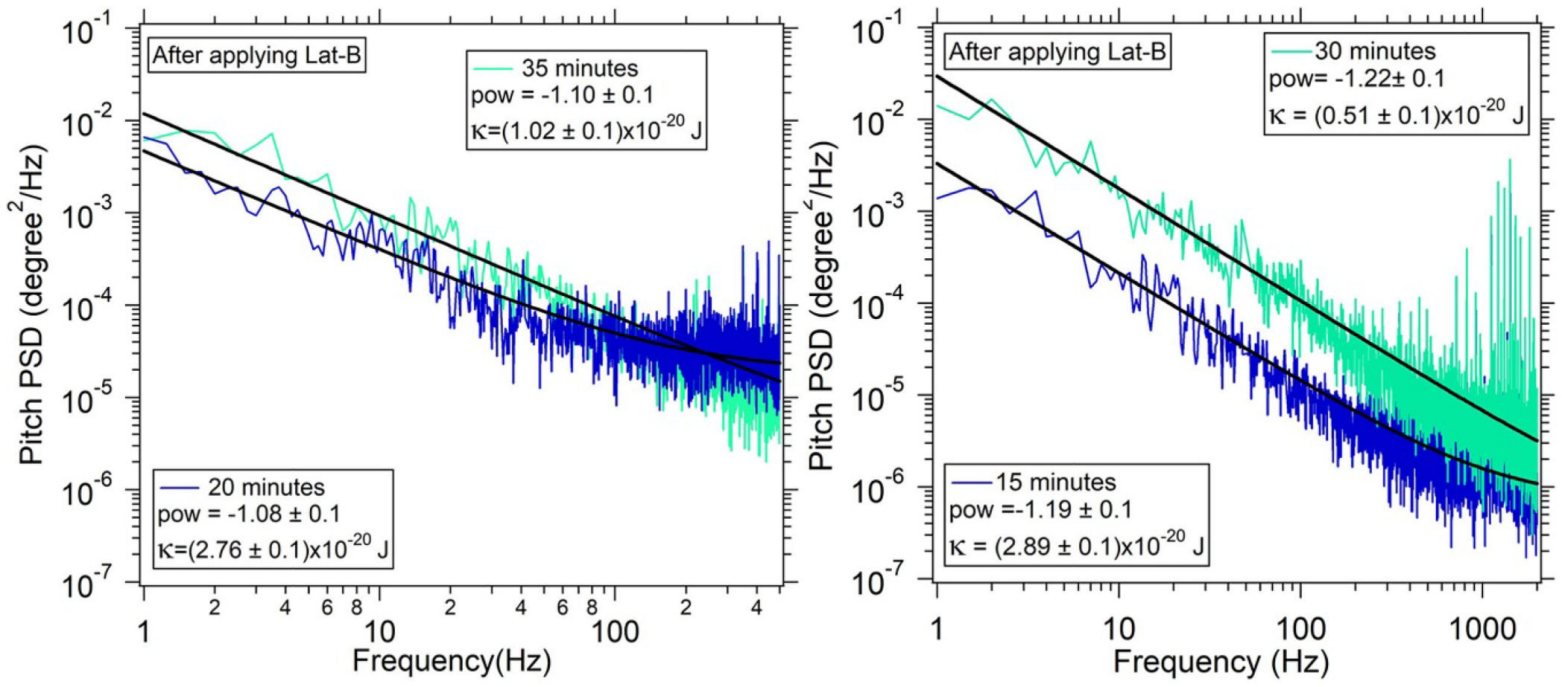
Two PSDs are recorded at an interval of 15 min after adding Latrunculin B with a concentration of (a)100 nM (b)200 nM A decrease in bending rigidity *κ* is seen at a later time(PSD in green).

We then compute the bending modulus from the coefficient of the PSD in equation (21). This has been shown in figure 5(b) for samples after application of Lat-B, at (4.6 ± 1.2) × 10^−20^ J. This can be compared with the bending rigidity obtained without Lat-B, reported in [31] at (1.9 ± 0.4) × 10^−19^ J. We estimate the softening of the MCF-7 cell membrane as a function of time, after applying Lat-B at 100 nM and 200 nM concentrations. Typical results are shown in figure 6. We find that the power law remains the same, albeit the coefficient changes. This implies that the bending rigidity in the 200 nM case changes by a factor of 6 from 2.8 × 10^−20^ J at 15 min after the application of the drug to 0.5 × 10^−20^ J at 30 min after the application of the drug. The results after this time were not reliable as we believe cells starts to die after 25–30 min from the time the sample has been placed on the optical tweezers set-up. The cells, attached to the glass surface, were placed on the optical tweezers unit at 15 min after application of Lat-B. Increase in amplitude of the PSDs by almost six times with time implies increased fluctuations with the onset of weakening of the actin cytoskeleton. With the actin cortex depolymerized, the cytoskeletal contribution to the membrane tension diminishes [48, 49]. Lower tension can be accounted by increased fluctuations. Figure 5(b) shows the calculated bending rigidities for 18 different events with a mean value of (4.6 ± 1.2) × 10^−20^ J which is close to values reported in other conventional methods [50–53].

We specifically compare our slope fluctuation method with the conventional technique of interference reflection microscopy (IRM) and find that IRM has an error of 30% [14]. Moreover, possibly due to the errors involved in the technique, particularly during the fitting of convoluted coefficients, the change in the bending rigidity without Lat-B and with Lat-B could not be made in IRM [14]. We can however clearly distinguish between the two cases, with a factor of 5 reduction in bending rigidity upon adding Lat-B. Our error in the measurement of the bending rigidity is about 10%, while the variation of the values of rigidity obtained is about 25%.

## 5 Conclusions

Thus, to conclude, we show the effect of the actindepolymerizing drug Lat-B on the PSD of the cell membrane fluctuations, both in the normal sense and in the pitch sense. We find that, even though the thermal fluctuations of the normal PSD at high frequency is expected to be dependent upon pinning effects, it is found independent in the case of the pitch PSD. Actin depolymerization reduces stiffening in this method which is reflected in reduction of the bending rigidity of the membrane. Measuring bending rigidity using this technique of slope fluctuation analysis is less complicated and more uniform over other methods such as tether pulling where results may vary depending on the tether pulling speed [53, 54] or other conventional methods like micropipette aspiration or AFM. We also look for active processes, particularly dependent upon actin polymerization, in the low frequency regime of the PSD but could not find any convincing evidence. Our technique could be used to probe active processes on the membrane, which however, may be a topic of further research but beyond the scope of the present manuscript.

## Data Availability

The data that support the findings of this study are available upon reasonable request from the authors.
